# Bioash-Based Stabilization/Solidification for Heavy Metal(oid) Soil Remediation: A Case Study in Northern Sweden

**DOI:** 10.3390/ma19040790

**Published:** 2026-02-18

**Authors:** Sepideh Gholizadeh Khasevani, Ivan Carabante, Josef Bjuhr, Lale Andreas

**Affiliations:** 1 Waste Science and Technology, Department of Civil, Environmental and Natural Resources Engineering, Luleå University of Technology, 97187 Luleå, Sweden; ivan.carabante@ltu.se (I.C.); lale.andreas@ltu.se (L.A.); 2 AFRY, Infrastructure AB, Frösundaleden 2A, 16970 Solna, Sweden; josef.bjuhr@afry.com

**Keywords:** bioash-based binder, cement reduction, soil stabilization, mechanical performance, field-scale validation

## Abstract

A bioash–cement composite binder was evaluated as a low-cement stabilization material for metal-contaminated soils, with emphasis on mechanical performance and long-term leaching behavior under field conditions. Two fine soil fractions from the Näsudden area (Skellefteå, Sweden), classified as hazardous (HS) and non-hazardous (NHS), were treated in laboratory trials to optimize binder composition. An optimum formulation containing 35 wt.% bioash and 5 wt.% cement (dry basis, relative to soil) improved unconfined compressive strength (UCS) to 696 kPa (HS) and 479 kPa (NHS) after 28 days and reduced leaching of Zn, Cd, Pb, and Co. Arsenic immobilization improved in HS but decreased in NHS, while Cu and Ni leaching increased, consistent with elevated pH and dissolved organic carbon (DOC) promoting soluble complexation. The optimized binder was then applied to a third soil (“Pilot soil”) and validated at pilot scale by treating 100 tonnes of soil and constructing a 2 m high noise barrier. Parallel laboratory tests on the Pilot soil yielded UCS values of 1000 kPa and confirmed effective retention of Zn and Cd, with generally good Pb stabilization, whereas As remained the most mobile element across soil types. Two-year field monitoring showed decreasing leachate concentrations of As, Cu, Ni, Pb, and Zn over time, and field samples exhibited improved Cu and Ni retention compared with laboratory results, suggesting progressive aging effects such as carbonation and mineral transformations. Overall, the results demonstrate that bioash–cement binders can produce mechanically stable treated materials suitable for low-load applications while reducing cement demand; however, performance is strongly controlled by soil-specific chemistry (notably DOC) and field execution (mixing and compaction), and further binder optimization is required to address arsenic mobility.

## 1. Introduction

Soil contamination by heavy metals poses a significant environmental challenge in northern Sweden, where historical mining and industrial activities have left a lasting legacy of pollution. The Näsudden area of Skellefteå exemplifies this issue, with soil contaminated by atmospheric emissions from Boliden’s smelter containing elevated concentrations of arsenic (403 mg/kg), copper (526 mg/kg), lead (806 mg/kg), and zinc (398 mg/kg). These persistent pollutants pose risks to both ecosystems and human health through potential leaching into groundwater and bioaccumulation [1,2,3].

Various technologies have been applied for soil remediation, including soil washing, phytoremediation, bioremediation, thermal desorption, and chemical oxidation [3,4,5,6]. However, most of these methods require excavation and off-site treatment or disposal, leading to high transport costs and large volumes of replacement material, while the contamination risk often remains [4,7]. At Näsudden, remediation is further complicated by uneven, rocky terrain and an estimated 100,000 tonnes of contaminated soil. Under such conditions, on-site treatment is more practical and cost-effective.

Stabilization and solidification (S/S) offer a promising in situ approach to metal-contaminated soils. The process works through two mechanisms: stabilization (adding binders to immobilize metals and prevent environmental migration) and solidification (creating a solid matrix to reduce contaminant mobility and accessibility) [8,9]. This dual approach simultaneously decreases pollutant solubility and improves soil mechanical properties. The technique reduces expenses related to soil removal and disposal, minimizes environmental impacts from transport activities, and maintains site integrity while effectively treating contamination [8,10,11]. As an on-site treatment, S/S provides multiple advantages: lower costs, reliable results, faster remediation time, elimination of soil transportation needs, and preservation of the original site. A wide range of materials including lime, cement, slag, polymers, and different types of ashes have been used as binders for soil stabilization [12,13,14,15]. Most binders, often derived from other industrial processes, offer a cost-effective and environmentally sustainable means of soil remediation [16,17]. This study evaluates a bioash-cement binder system designed to reduce cement consumption while maintaining adequate soil strength and effective metal immobilization. While cement remains the most common stabilization additive, its use presents significant drawbacks: high CO_2_ emissions (production of cement accounts for ~8% of global emissions), increased soil pH, cracking risks, and long curing times [18,19,20].

Ashes are residual materials from combustion processes, such as coal or biomass burning. They can contain high amounts of heavy metals and other contaminants, making proper management essential to prevent environmental contamination [21,22,23]. According to various studies, researchers show that metal leaching from ash-stabilized soil can decrease significantly, potentially by over 90% [21,23]. In stabilization applications, ashes function as binders due to their pozzolanic properties, enabling reactions with calcium hydroxide in water to form calcium silicate hydrate (C-S-H) and calcium aluminate hydrate (C-A-H). These compounds enhance soil strength, durability, and load-bearing capacity while reducing permeability, making ashes effective and eco-friendly alternatives [24,25,26]. Bioash, derived from wood and agricultural residue combustion, exhibits favorable physical properties for stabilization/solidification (S/S), including small particle size (increasing reactive surface area), high density (improving compactness), and porosity (facilitating contaminant absorption) [27,28]. Chemically, bioash contains Ca, Fe, Al, Si, and Mn oxides, which form insoluble compounds with hazardous metals [16,29]. Despite these advantages, bioash alone lacks sufficient hardening properties for effective S/S [30,31] and must be combined with cement, slag, or other strengthening agents to achieve robust mechanical performance and contaminant immobilization [15,30,31]. The S/S process relies on binder-water reactions that release hydroxide ions, elevating pH and creating multiple immobilization mechanisms. This alkaline environment promotes CSH gel formation for structural stabilization while simultaneously reducing heavy metal solubility through precipitation as stable, insoluble compounds. The pH increase also modifies soil surface chemistry, enhancing adsorption of cationic contaminants and significantly reducing leaching potential [32,33,34]. Together, these mechanisms, physical encapsulation through cementitious products, chemical precipitation of metals, and enhanced surface adsorption make bioash-enhanced S/S an effective approach for comprehensive soil remediation. The S/S process creates a durable solidified matrix that significantly improves the treated material’s structural integrity and long-term stability. This enhanced performance makes the stabilized material suitable for multiple practical applications, including use as landfill cover layers or as components in construction materials [19,35,36]. Most existing investigations of bioash-based binders have been limited to laboratory-scale experiments conducted under controlled curing conditions. While such studies are essential for binder formulation and mechanistic understanding, they do not fully capture the effects of field processing, including large-scale mixing, compaction variability, moisture heterogeneity, and environmental exposure. From a materials engineering standpoint, field-scale validation is essential to assess whether laboratory-optimized binders retain their mechanical integrity and leaching performance under realistic service conditions and over extended time periods.

Despite extensive research on cement-based stabilization/solidification of contaminated soils, most studies have remained limited to laboratory-scale testing and have focused primarily on short-term performance using high cement contents [22,37]. There is a lack of integrated studies that combine laboratory optimization with large-scale field application and long-term monitoring, particularly for low-cement binder systems incorporating industrial by-products such as bioash. In addition, the influence of soil-specific properties, especially dissolved organic carbon (DOC), on stabilization performance has remained insufficiently understood under realistic field conditions. This study addresses these gaps by hypothesizing that bioash–cement binders can be optimized to reduce cement consumption while maintaining adequate mechanical strength and long-term immobilization performance, provided that soil chemistry is explicitly considered.

Against this background, this study focused on developing and validating an optimized bioash–cement binder recipe for the S/S of metal-contaminated soils. The work progressed from laboratory-scale optimization to full-scale field implementation, with three main objectives: (1) to identify the optimal bioash–cement ratio for achieving both effective contaminant immobilization and adequate mechanical strength, (2) to assess binder performance under real field conditions through the construction of a noise barrier involving approximately 100 tonnes of treated soil compacted into a 2 m high structure, and (3) to evaluate long-term effectiveness through nearly two years of post-treatment monitoring. This study is one of the few to combine laboratory-scale optimization with large-scale field application and long-term monitoring of bioash-based binders, providing insights into the real-world performance of these systems and highlighting the role of soil-specific properties such as dissolved organic carbon (DOC).

In the first phase, two contaminated soils classified as hazardous soil (HS) and non-hazardous soil (NHS) were treated using different binder formulations (65:35:0, 60:35:5, 50:50:0, and 47.5:47.5:5 soil:bioash:cement, dry weight) to identify the most effective mixture based on trace element immobilization and unconfined compressive strength (UCS). The optimal formulation was subsequently applied to the Näsudden soil (“Pilot soil”) and evaluated at both laboratory and pilot scales. This real-world application provided valuable insight into the long-term stability, environmental performance, and scalability of the bioash–cement binder system. By linking binder composition, mechanical performance, chemical stability, and field-scale durability, this work contributes to the development of low-cement, bioash-based binders for low-load construction applications and sustainable soil remediation.

## 2. Materials and Methods

### 2.1. Raw Materials and Soil Substrates

Three fine-grained soil substrates were used to develop and evaluate the bioash–cement binder system for stabilization/solidification (S/S). Two soils were obtained from an area adjacent to the Näsudden site (Skellefteå, Sweden) and processed at a materials handling facility using the same sieving equipment and particle size threshold (≤10 mm) planned for the pilot-scale application. Based on total trace element concentrations, these soils were classified as hazardous soil (HS) and non-hazardous soil (NHS), respectively (Table 1). The HS and NHS were selected for laboratory-scale binder formulation because their contaminant levels were comparable to, or higher than, those expected in the full-scale application, thereby providing a conservative basis for material performance evaluation. A third soil, referred to as the Pilot soil, was excavated directly from the Näsudden remediation site. The material comprised a mixture of organic and mineral soil layers collected from a depth of 0–0.4 m. After excavation, the soil was sieved at the same materials handling facility using the identical procedure applied to HS and NHS to isolate the fine fraction. Approximately 80 kg of the sieved Pilot soil was homogenized using a riffle splitter and stored in sealed containers prior to laboratory testing and characterization. The total concentrations of trace elements in HS, NHS, and the Pilot soil, as well as in the binder constituents (bioash and cement), are summarized in Table 1. The HS represents a highly contaminated material, while the NHS serves as a moderately contaminated reference. The Pilot soil exhibited similarly elevated concentrations of As and Pb, allowing the binder system to be evaluated across a range of realistic contamination scenarios and providing insight into soil-dependent leaching behavior and binder performance. The soil types investigated and their abbreviations, regulatory classifications, and applications in laboratory and field experiments are summarized in Table 1. The particle size distributions of the soils (HS, NHS, and Pilot soil) are summarized in the Supporting Information (Figure S1), where it is shown that all the soils consist primarily of coarse particles, isolated using sieving at ≤10 mm.

The bioash used as the primary supplementary binder component was a wood ash supplied by Skellefteå Kraft (Skellefteå, Sweden). Although delivered in dry form, the ash had been temporarily stored under roofed but open conditions and was therefore potentially exposed to ambient moisture. To ensure consistent material properties and prevent premature reactions prior to testing, approximately 80 kg of ash was dried at 40 °C in a ventilated cabinet for 2–4 days and stored under dry conditions until use. The bioash is predominantly fine-grained, with nearly complete passing at the smallest sieve use (≤0.5–1 mm), corresponding to silt and clay range. This fine, powdery nature enhances its reactivity, making it suitable for stabilization/solidification (S/S) applications. The particle size distribution is provided in the Supporting Information (Figure S1).

Ordinary Portland cement, used as the primary hydraulic binder component in all formulations, was supplied by Cementa AB (Slite, Sweden), part of the HeidelbergCement Group.

The chemical (oxide) composition of the Pilot soil and binder materials is provided in the Supporting Information (Table S1).

### 2.2. Binder Formulation and Specimen Preparation

Stabilized specimens were prepared by blending soil substrates with controlled proportions of bioash, cement, and water to form bioash–cement composite binders. Four binder formulations were investigated in laboratory-scale trials, expressed as soil:bioash:cement ratios by dry weight: 65:35:0, 60:35:5, 50:50:0, and 47.5:47.5:5. These mixtures were selected to evaluate the influence of bioash content and partial cement substitution on mechanical performance and leaching behavior. The selected binder ratios were designed to reflect realistic stabilization scenarios while prioritizing reduced cement usage. Formulations without cement were included to assess the standalone stabilization potential of bioash. Mixtures containing a low cement fraction (5 wt.%) were selected to evaluate the minimum cement addition required to enhance early strength development and alkalinity. The investigated bioash contents were chosen to balance workability, material availability, and feasibility of large-scale mixing, ensuring that the formulations remained applicable under field conditions rather than optimized solely for laboratory performance. However, formulations with lower bioash ratios resulted in poor compaction and insufficient binder strength under field-relevant conditions. Therefore, these formulations were not considered ideal for practical implementation. The selected formulations were designed to ensure adequate compaction and mechanical stability, which are essential for leaching control and durability in real-world applications. For each formulation, the required masses of soil, bioash, and cement were accurately weighed and dry-mixed until a visually homogeneous blend was obtained. Deionized water was then gradually added while mixing continuously to ensure uniform moisture distribution. The target water content for each mixture was determined based on the corresponding optimum moisture content obtained from Proctor compaction tests, thereby simulating realistic field compaction conditions and minimizing variability in specimen density. The Pilot soil was treated using the laboratory-optimized formulation containing 35 wt.% bioash and 5 wt.% cement (relative to dry soil). Specimens were prepared following the same mixing procedure used in the laboratory trials, with water added incrementally to achieve a moisture content of approximately 15%, corresponding to optimal compaction conditions for this material. For all experimental investigations, including unconfined compressive strength (UCS) testing, batch leaching tests, and diffusion leaching tests, replicates were prepared and analyzed for each soil–binder formulation. Reported values are presented as mean ± standard deviation, reflecting variability between replicate measurements. This variability results from differences that can occur due to slight variations in moisture content, compaction techniques, and binder mixing methods, which are inherent in real-world applications and large-scale operations.

### 2.3. Characterization of the Samples

The XRD diffractograms of the degassed samples were collected in the 2θ range from 5° to 120° using a Cu Kα radiation X-ray diffractometer (XRD, PANalytical Empyrean X-ray diffractometer, Malvern, UK).

### 2.4. Test Methods

**Total Solids (TS) and Loss On Ignition (LOI)**

The TS and LOI were analyzed following the Swedish Standard SS 28113 [38].

**X-Ray Fluorescence (XRF)**

The chemical compositions of soil, bioash, and cement were analyzed using X-ray fluorescence (XRF) spectroscopy (Thermo Scientific Niton XL3t, Waltham, Billerica, MA, USA). Each sample was homogenized before analysis, with three replicate measurements taken to ensure data reliability.

**Compaction**

Compaction characteristics of the soil-binder mixtures were determined using a standard Proctor compaction test in accordance with SS-EN 13286-2:2010/AC:2013c [39]. The test was used to establish the optimum water content (OWC) and maximum dry density (MDD) for each mixture. Compaction was performed in a cylindrical mold in three layers, with each layer compacted by five drops of a 2 kg rammer falling from a height of 20 cm, corresponding to a compaction energy of approximately 550 kJ/m^3^. After compaction, the wet mass of the sample was recorded, and a portion was oven-dried to determine its dry mass and calculate the moisture content.

**Unconfined compressive strength (UCS) test**

Unconfined compressive strength (UCS) testing was performed following the procedures specified in the Swedish standard SS 27128. After the designated curing periods, specimens were subjected to axial compression until failure. The peak stress achieved during loading was taken as the UCS value and used to assess strength development as a function of curing time. Six specimens were prepared during the construction of the pilot-scale noise barrier. These test tubes were compacted on site using the same Proctor hammer as in the laboratory. Tubes 1–3 were filled with material from the first batch used in the noise barrier, while tubes 4–6 were filled with material from a third batch in which the water content had been increased. Due to the still lower moisture content compared to lab mixtures, and because the field setup did not allow for fully optimized compaction equipment, the field specimens were compacted using increased packing energy, applied as a higher number of blows per layer than in the lab (nine vs. five). To evaluate the influence of this increased compaction effort under controlled conditions, additional UCS tests were later conducted in the laboratory using the same modified compaction procedure. For UCS measurements, two replicates (*n* = 2) were performed for each binder formulation for NHS and HS, and three replicates (*n* = 3) were performed for Pilot Soil under both laboratory and field conditions.

**Batch Leaching Tests (L/S = 10)**

To evaluate element immobilization, a batch leaching test was performed following the Swedish standard SS-EN 12457-2. Triplicates of each recipe were prepared for testing. The cured specimens were crushed before leaching and mixed with deionized water as the leachant at a liquid-to-solid (L/S) ratio of 10 L/kg. The particle size after crushing was <4 mm. During the 24 h agitation by overhead rotation, continuous friction—both between particles and against the container walls—enhanced element release. Finally, the suspensions were filtered through 0.45 μm membrane filters.

Leaching results are reported as mass released per unit dry mass of soil (mg kg^−1^), normalize contaminant release to the solid matrix and allow consistent comparison across tests with different liquid-to-solid ratios.

**Diffusion Leach Tests**

Cumulative element release and leaching mechanisms were assessed through diffusion tests on the optimal binder mixture. Leaching behavior was interpreted using slope analysis according to NEN 7345 to determine controlling mechanisms on the triplicates of the optimal recipes (35% bioash and 5% cement) for both HS and NHS. The mixtures were moistened with 10% distilled water (optimum water content for compaction) and mixed thoroughly. Samples were prepared by packing soil and binder mixtures into plastic tubes (height = 50 mm, diameter = 50 mm), following the same compaction as for the UCS test. For testing, each plastic tube was placed in a plastic bottle containing 0.7 L of deionized water. The experiment spanned 64 days. During this time, water samples were collected from all bottles at eight points: 6 h, 24 h, 54 h, and days 4, 9, 16, 36, and 64. At each sampling, the bottles were fully emptied. The collected water samples were filtered using 0.45 µm membrane filters.

The cumulative leached amount per unit surface area was determined using Equation (1):

(1) $$B(t)=\frac{\sum_{i=1}^{n}C_{i}V_{i}}{A}$$

where B(t)(mg m^−2^) is the cumulative leached amount at time t, $C_{i}$(mg L^−1^) is the concentration measured in the leachate at interval i, $V_{i}$(L) is the volume of leachant collected at interval i, A(m^2^) is the exposed surface area of the specimen, and n is the number of leaching intervals.

**Chemical analysis**

The pH and electrical conductivity (EC) of the eluates were measured directly after filtration through 0.45 mm membrane filters. Samples were then stored in the refrigerator until further analysis. Total organic carbon (TOC) was analyzed using a TOC-V CSH analyzer (Shimadzu Corporation, Kyoto, Japan). Element concentrations in the eluates were analyzed by inductively coupled plasma atomic emission spectroscopy (ICP-AES).

## 3. Pilot Test

### 3.1. Description of the Pilot Site

The Näsudden site is located in Skelleftehamn, northern Sweden, directly north of the Boliden Rönnskär smelter. The area, covering approximately 70 hectares, has been impacted by historical atmospheric emissions from smelting operations, resulting in widespread soil contamination with arsenic (As), lead (Pb), copper (Cu), and zinc (Zn). The contaminants are primarily concentrated in the organic-rich topsoil (approximately 0–0.1 m depth), with declining concentrations in the underlying mineral layer (0.1–0.3 m). Investigations have shown relatively homogeneous contamination across the site, with total soil burdens estimated at approximately 46 tonnes of arsenic and 90 tonnes of lead. Trace element concentrations in the untreated soil used for the pilot application are provided in Table 2, confirming that As, Cu, and Pb exceeded the guideline values for less sensitive land use.

The site is planned for future industrial development, and remediation is required to manage risks associated with soil contact and contaminant migration. Conventional remediation methods such as excavation and off-site disposal are impractical due to the site’s rocky terrain, shallow but extensive contamination, and the large volume of affected soil—estimated at approximately 100,000 tonnes. As a more sustainable alternative, an on-site stabilization/solidification (S/S) approach was selected to minimize soil disturbance, reduce transport and disposal needs, and support beneficial reuse of treated material.

**Figure 1:**
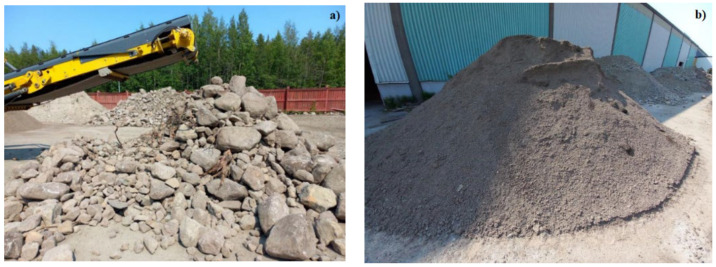
(**a**) Sorted coarse fraction from test box, (**b**) Sorted fine fraction from test box 2. A total of approx. 100 tonnes of contaminated fine fractions were obtained during the sorting process.

To evaluate the field performance of the S/S method, a pilot-scale test was carried out in June 2023. Approximately 100 tonnes of contaminated fine-fraction soil (0–0.4 m depth) was excavated and sieved to <10 mm (Figure 1). This material was treated with a binder mixture consisting of 35% bioash and 5% cement (dry weight), with water added to achieve a moisture content of approximately 10–15%. The bioash was supplied by Skellefteå Kraft and the cement by Cementa AB. The treated soil was compacted in layers to construct a 2 m high noise barrier along an internal access road within the site.

The barrier was designed to fulfill both a technical function—noise reduction—and a demonstration role, providing a field-scale representation of how stabilized material could be implemented on site. The field trial included detailed documentation of mixing and compaction procedures, installation of test specimens, and a leachate collection system. Subsequent monitoring over a two-year period was conducted to assess mechanical integrity, contaminant mobility, and environmental performance under realistic field conditions.

### 3.2. Construction and Monitoring of the Noise Barrier

In June 2023, about 100 tonnes of contaminated fine-fraction soil was stabilized using the selected binder mixture of 35% bioash and 5% cement (dry weight), with 16 m^3^ of water added to achieve the target moisture content. The bioash was supplied by Skellefteå Kraft and the cement by Cementa AB. The contaminated soil and dry binders were initially pre-mixed using scoop mixing at ground level (Figure S2a–c). The blended material was then loaded onto a truck and transported from the mixing area to the pilot site. This loading and unloading process contributed additional mechanical mixing. On site, the moistened binder–soil mass was further prepared in a mixing pit (Figure S2d), where water was added via a hose with flow-meter control. Final mixing was conducted with an aluminum scoop during placement on the barrier structure (Figure S2e).

The noise barrier was constructed in layers, each compacted using a 250 kg vibratory plate compactor. The base dimensions were 16 m (length) by 5 m (width), and the structure was built to a height of 2 m. Due to side slope construction, the top of the barrier measured approximately 12 m in length and 1 m in width (Figure S2f). In parallel with construction, cylindrical test specimens were prepared for strength analysis. Seven test tubes were installed—three from the first mixing batch and four from the second—compacted manually in three layers with a 2 kg hammer, using nine drops per layer to achieve approximately 20 cm lift per sample. Specimens were cured in sealed containers under moisture-saturated conditions for 28 days prior to UCS testing.

To monitor leaching behavior, the noise barrier was constructed on a heavy-duty tarpaulin laid with a natural slope directing infiltrated water into a buried 200 L collection container (Figure S3). The system captured percolating leachate and surface runoff. In parallel, a separate bucket mounted in a nearby tree was used to collect rainwater for comparison.

### 3.3. Monitoring Pilot Performance: Field Observations and Sampling

To evaluate the performance of the pilot application, leachate was monitored over the course of two years, and solid samples were collected once, two months after construction of the noise barrier. Initial leachate sampling was conducted one month after implementation, and subsequent monitoring took place at intervals dictated by rainfall events, which were necessary to enable leachate generation. Due to the long winter period in northern Sweden, field sampling was not possible for several months, and monitoring resumed with the return of milder conditions and precipitation in spring.

Leachate was retrieved from the 200 L collection container positioned beneath the tarpaulin under the barrier. A peristaltic pump was used to extract samples, and parameters such as pH and electrical conductivity (EC) were measured immediately after collection. For each sampling occasion, six leachate samples of 50 mL were taken, three of which were filtered through 0.45 µm syringe filters (Figure S4a,b). To ensure discrete sampling, the container was fully emptied between events. Samples were collected to analyze trace elements using ICP and DOC analysis.

Rainwater was collected in a bucket placed on a tree stump to avoid contact with leaves and ensure sampling of direct atmospheric deposition. Precipitation data for the monitoring period were obtained from the Swedish Meteorological and Hydrological Institute (SMHI) to support interpretation of rainfall input volumes. Solid samples from the noise barrier were collected two months after construction. Sampling was carried out at four locations (1–4) around the structure, with two depth intervals at each location: 0–5 cm (layer a) and 10–20 cm (layer b). Total metal content in the solids was analyzed using X-ray fluorescence (XRF), and additional leaching tests were performed to assess element release from the barrier material under standardized conditions.

## 4. Results and Discussion

### 4.1. Composition of Soils and Binder Materials

The total content of trace elements in the untreated HS and NHS, the Pilot soil, and the binders (bioash and cement), along with total solids (TS), volatile solids (VS), and total organic carbon (TOC), is presented in Table 2. Each category’s corresponding threshold values for these elements are highlighted to assess soil contamination levels and to understand the implications of land use on contamination control.

The contaminated soil samples (HS and NHS) contain elevated concentrations of several trace elements. Specifically, As, Cu, and Pb concentrations in these samples exceed the Swedish guideline values for both sensitive and less sensitive land. Arsenic levels in HS (168 mg/kg) and NHS (102 mg/kg) are above the SL threshold (10 mg/kg), highlighting the soil’s contamination. Copper and lead concentrations in both samples are also significantly higher than the SL limits.

The Pilot soil, excavated in June 2023, shows a moderate reduction in metal concentrations compared to HS and NHS, but still contains elevated levels of certain elements, notably Cu and As. Arsenic in Pilot soil is higher than in HS (168 mg/kg) and NHS (102 mg/kg), exceeding the SL threshold of 10 mg/kg. Copper in Pilot soil is higher than in HS (266 mg/kg) and NHS (160 mg/kg), exceeding the SL threshold of 200 mg/kg. Zinc in Pilot soil is lower than in HS (548 mg/kg), but higher than in NHS (240 mg/kg), remaining below the SL threshold of 500 mg/kg. The bioash used in the lab trials contained element concentrations below the LSL thresholds for all elements except As, and approached the SL limits for Cu and Cr. In the cement, all elements were below detection limits except for Pb and Zn. The Pb content (31.25 mg/kg) was below the LSL limit, while Zn (1026 mg/kg) exceeded it.

Table S1 presents the major metal oxide composition of the binders and the contaminated soil. The Pilot soil is characterized by a relatively high silicon dioxide (SiO_2_) content (67.8 wt.%), with calcium oxide (CaO) at 2.47 wt.%, which is important for its physical properties but not primarily reactive in cementitious processes. The bioash is characterized by a relatively high calcium oxide (CaO) content (33.7 wt.%), which is a key component for cementitious reactions and alkalinity generation during the stabilization/solidification (S/S) method. In addition, bioash contains appreciable amounts of silicon dioxide (SiO_2_, 24.1 wt.%) and aluminum oxide (Al_2_O_3_, 5.96 wt.%), which can participate in pozzolanic reactions and contribute to the formation of secondary binding phases over time. These combined properties make bioash a suitable candidate for use as a low-cement binder in S/S applications.

Portland cement exhibits a strongly Ca-rich composition, with CaO accounting for 63.3 wt.%, while SiO_2_ (21.2 wt.%), Al_2_O_3_ (3.4 wt.%), and Fe_2_O_3_ (4.1 wt.%) are present at lower levels. The high CaO content of cement is primarily responsible for rapid development and pH increase, which promote precipitation, sorption, and incorporation of contaminants into cementitious matrices. The combination of Ca-rich binders (bioash and cement) with SiO_2_- and Al_2_O_3_-bearing phases supports both short-term cementitious reactions and longer-term pozzolanic processes. These mechanisms contribute to the stabilization and solidification of metal contaminants through pH control, matrix densification, and incorporation of elements into low-solubility solid phases, thereby enhancing both mechanical stability and leaching resistance of the treated soils.

### 4.2. XRD Analysis of Bioash, Cement, and (60%Pilot Soil:35%A:5%C) Mixture

**Figure 2:**
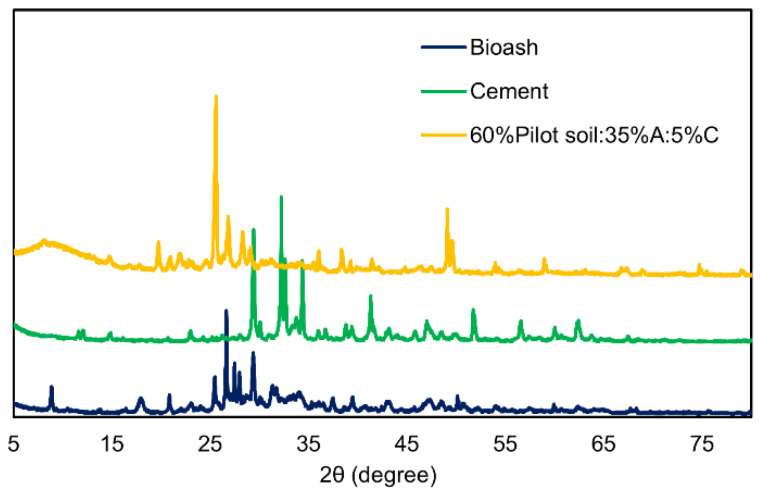
XRD patterns for bioash, cement, and a 60% pilot soil:35% bioash:5% cement mixture (60%Pilot soil:35%A:5%C, as best recipe).

Figure 2 shows the X-ray diffraction (XRD) patterns for bioash, cement, and the 60% Pilot soil: 35% Bioash: 5% Cement mixture. The XRD pattern of bioash displayed prominent peaks for SiO_2_ (quartz) around 2θ = 25°, CaCO_3_ (calcite) at 2θ = 29°, and Fe_2_O_3_ (iron oxide) at 2θ = 36°, indicating the presence of common ash minerals. Smaller peaks at 2θ = 34° and 2θ = 50° corresponded to Al_2_O_3_ (aluminum oxide) and CaO (calcium oxide), respectively, suggesting cementitious potential [41,42]. The XRD pattern of cement revealed the presence of C3S (Ca_3_SiO_5_), C2S (Ca_2_SiO_4_), C3A (Ca_3_Al_2_O_6_), C4AF (Ca_4_Al_2_Fe_2_O_12_), and CaSO_4_, along with amorphous C-S-H phases. The key peaks observed were at 2θ ≈ 29°, 32°, 34° (C3S), 2θ = 30° (C2S), 2θ =15° and 30° (C3A), 2θ =18° and 27° (C4AF), and 2θ = 11° and 20° (CaSO_4_), phases that together contribute to the material’s hydration, strength development, and setting properties [43].

The XRD pattern for the mixture showed overlapping peaks from both bioash (mainly SiO_2_) and cement (C_3_S, C_3_A, and C_4_AF), suggesting that both materials were present in the mixture. Although no distinct peaks for C-S-H (calcium silicate hydrate) or C-A-S-H (calcium alumino-silicate hydrate) were observed in the 2θ range of 29–35°, which are typically indicative of hydration reactions, the pattern suggests that no significant crystalline phases have formed from the interaction between bioash and cement. This may imply that the reaction between these two components is still in its early stages or that major crystalline phases did not form under the conditions tested [44].

### 4.3. Compaction Behavior and Strength Development

The compaction behavior and mechanical performance of all stabilized soils were evaluated based on Proctor test results (optimum water content and maximum dry density) and unconfined compressive strength (UCS). Results were compared across HS, NHS, and Pilot soils under both laboratory and field conditions. Differences in UCS development are interpreted in the context of binder formulation, compaction efficiency, and moisture content.

Table S2 presents the optimum water content (OWC) and maximum dry density (MDD) for each binder formulation tested with HS, NHS, and Pilot soil. For the recipe with 35% bioash and 5% cement (35%A:5%C), the OWC was 10% for both HS and NHS. Increasing the bioash content to 47.5% (47.5%A:5%C) raised the OWC to 21% for both soils. While identical OWC values across these two soil types might seem unexpected, no clear explanation can be drawn based on the available data vs. content was slightly higher in NHS (9%) than in HS (5.6%), which could indicate greater organic content and potentially higher water demand, but this trend was not reflected in the OWC results. Other factors such as particle size distribution or microstructural differences may have influenced the compaction behavior, though such data were not available in this study [45].

For the Pilot soil using the same 35%A:5%C mixture, the OWC was 15%, notably higher than for HS and NHS. This result likely reflects differences in the physical properties of the Pilot soil, such as its texture, mineral-organic composition, and total solids content, which increased its water requirement to achieve maximum compaction.

**Unconfined compressive strength (UCS)**

**Figure 3:**
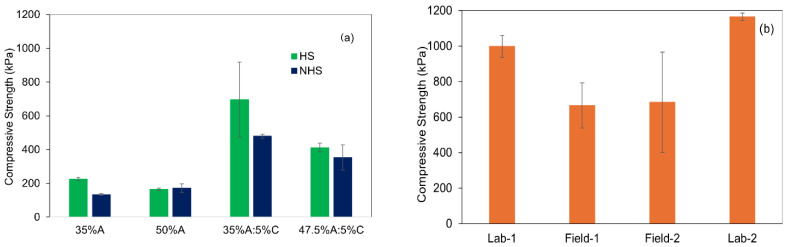
(**a**) Compaction of HS and NHS under varying compaction conditions in laboratory tests. (**b**) Compaction of 60% Pilot Soil:35% Bioash:5% Cement under varying compaction conditions in lab and field tests. Lab 1: Standard Proctor Test; Lab 2: Heavy compaction with increased energy (9 blows per layer compared to standard compaction). Field 1 and Field 2: Heavy compaction in the field.

Figure 3a (HS and NHS) and Figure 3b (Pilot soil) show the unconfined compressive strength (UCS) of the stabilized soil mixtures after curing for 28 days. For HS and NHS, the highest UCS values are achieved with the formulation containing 35% bioash and 5% cement, reaching 696 ± 222 kPa for HS and 479 ± 10.82 kPa for NHS. HS consistently produced slightly higher strength than NHS across all binder formulations. The addition of 5% cement increases UCS by approximately three times compared to bioash-only mixtures, confirming the essential role of cement in enhancing mechanical stability. The strength gain is attributed to hydration and pozzolanic reactions forming calcium silicate hydrate (C-S-H) and calcium aluminate hydrate (C-A-H) gels, which densify the matrix and improve cohesion [25,26].

The same binder formulation was applied to the Pilot soil. Laboratory testing showed a UCS of 999 ± 62 kPa under standard compaction, and 1165 ± 21 kPa with increased compaction energy. Field-compacted samples reached an average UCS of 666 ± 127 kPa (first batch) and 683 ± 282 kPa (second batch with higher water content), lower than in the lab but still within a usable range. The reduced strength in the field is attributed to less uniform mixing, variations in moisture content, and suboptimal compaction equipment.

Observations during and after construction of the noise barrier revealed that the upper surface remained soft, indicating insufficient compaction. The barrier was constructed in layers, with the lower layers compacted using a 250 kg vibratory plate compactor. However, as the structure grew in height, the upper layers were compacted only with the excavator bucket, which provided significantly less compaction energy. This variation in compaction method likely contributed to reduced mechanical stability at the surface and highlights the challenge of ensuring consistent compaction quality under field conditions.

While the UCS values from field-compacted test specimens still fell within the range commonly reported for compacted clay, weak concrete, or soft sedimentary rock, this indicates that the material is mechanically suitable for low-load applications such as noise barriers or engineered fill. However, the soft surface texture of the constructed barrier suggests that binder performance alone is not sufficient. Achieving reliable field performance also requires proper moisture content, consistent layer-wise compaction, and the use of suitable equipment throughout the construction process.

**Field UCS vs. Laboratory UCS**

The UCS values measured in the pilot-scale construction were consistently lower than those obtained from laboratory-prepared specimens. This discrepancy can be attributed to differences in preparation and curing conditions. Laboratory specimens were produced under controlled moisture content, homogeneous mixing, and uniform compaction energy. In contrast, field stabilization involved heterogeneous soil conditions, variable moisture distribution, and less controlled compaction, which often result in reduced density and bonding efficiency.

Field compaction methods were less effective than laboratory methods, particularly in terms of compaction energy. For instance, the upper layers of the constructed barrier were compacted using an excavator bucket, which provided significantly less compaction energy than the 250 kg vibratory plate compactor used for the lower layers. This variation in compaction technique likely contributed to the reduced mechanical stability at the surface.

**Factors affecting Field UCS:**

1. Moisture Variability: In the field, moisture content is harder to control than in laboratory tests, and moisture variations can impact both the compaction process and the formation of C-S-H and C-A-H gels, which are critical for strength development.
2. Compaction Efficiency: Differences in compaction energy and uniformity can lead to inconsistent strength development. Equipment such as excavator buckets may not provide the same level of compaction efficiency as standard laboratory or vibratory plate compactors.
3. Field Soil Conditions: Field soil is often heterogeneous, unlike the uniform soil used in lab tests. Variations in soil texture, organic content, and particle size distribution can influence how the binder interacts with soil particles, which in turn affects UCS results.

**Statistical Interpretation of UCS Test Results**

No statistically significant difference was observed between the UCS values of HS and NHS using the different binder formulations (*p* = 0.55, *p* > 0.05). Both soils demonstrated similar mechanical performance with this specific binder formulation. However, a statistically significant difference was found between lab-compacted and field-compacted Pilot Soil (*p* = 0.039, *p* < 0.05). Lab compaction consistently resulted in higher UCS values compared to field compaction, suggesting that field compaction conditions, such as moisture variability and inconsistent mixing, negatively impacted the UCS.

### 4.4. Element Mobility and Stabilization Performance

The effectiveness of the stabilization/solidification treatment was assessed by evaluating the leaching behavior of key trace elements under laboratory and field conditions. This section summarizes the results of batch leaching, diffusion tests, and field monitoring.

#### 4.4.1. Batch Leaching Tests (L/S = 10)

**Figure 4:**
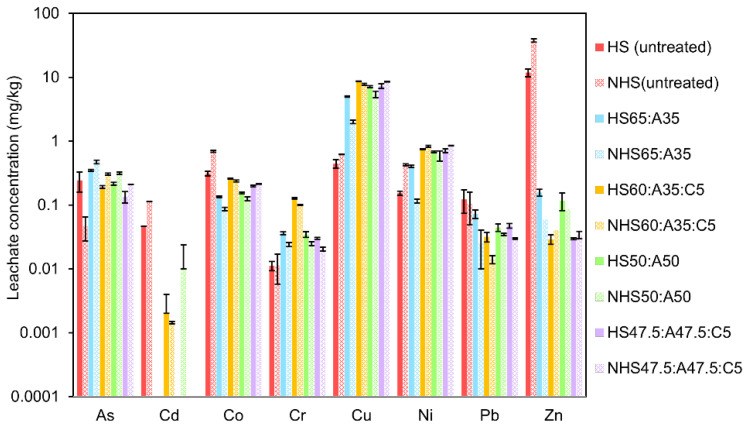
The leaching from the HS and NHS before and after stabilization at L/S = 10.

Results from the batch leaching tests (L/S = 10) for the HS and NHS fine fractions before and after stabilization are summarized in Table S3 and Figure 4. The eluates from the untreated soils exhibit slightly acidic pH values with 6.4 for HS and 5.5 for NHS. After stabilization, all binder formulations produce a pronounced increase in pH, with values ranging from 9.6 to 12.3. The highest pH levels are observed for mixtures containing 47.5 wt.% bioash and 5 wt.% cement in both soils, reflecting the strong alkalinity introduced by the binder components.

Electrical conductivity (EC) shows distinct trends depending on binder composition. Prior to treatment, NHS exhibits a higher EC (7.0 mS cm^−1^) than HS (4.1 mS cm^−1^), likely reflecting differences in the native ionic composition of the soils, including soluble salts and metal species. Following stabilization with bioash alone, EC unexpectedly decreases, from 4.1 to 2.6 mS cm^−1^ in HS and from 7.0 to 5.3 mS cm^−1^ in NHS for the 35 wt.% bioash mixture. This decrease is attributed to precipitation reactions under alkaline conditions and/or sorption of dissolved ions onto ash-derived mineral phases.

When cement is included in the binder formulations (35%A:5%C and 47.5%A:5%C), EC increases again, reaching 3.8 mS cm^−1^ in HS and 7.2 mS cm^−1^ in NHS. This increase reflects the release of hydroxide and other alkaline ions during cement hydration, which elevates the ionic strength of the pore solution. The combined use of cement and bioash therefore results in higher EC values than bioash alone, particularly in NHS, where the initial ionic strength is already elevated.

Dissolved organic carbon (DOC) concentrations increase markedly following stabilization, especially in mixtures containing both bioash and cement. The highest DOC concentrations are measured for the 35%A:5%C formulation, reaching 96.9 mg L^−1^ in HS and 146.5 mg L^−1^ in NHS. This increase is attributed to enhanced solubilization of native soil organic matter under alkaline conditions, as well as the contribution of soluble organic compounds originating from the bioash. Elevated DOC levels often coincide with higher EC values, suggesting that organic ligands contribute to the overall ionic load. This behavior is consistent with the formation of soluble organo–metal complexes involving dissolved organic carbon and alkaline metals or salts.

The leaching behavior of trace elements before and after stabilization is illustrated in Figure 4. The untreated HS and NHS exhibit distinct leaching patterns. NHS releases higher concentrations of Cu, Zn, Ni, and Cr, whereas HS shows greater leaching of As and Pb. For example, Zn concentrations reach 37 ± 2.5 mg/kg in NHS compared to 11.8 ± 1.65 mg/kg in HS, and Ni concentrations reach 0.42 ± 0.015 mg/kg in NHS versus 0.15 ± 0.01 mg/kg in HS. In contrast, As concentrations are higher in HS (0.24 ± 0.085 mg/kg^−1^) than in NHS (0.046 ± 0.019 mg/kg), as are Pb concentrations (0.12 ± 0.048 mg/kg in HS compared with 0.1 ± 0.05 mg/kg in NHS).

These differences in leaching behavior are consistent with the lower pH, higher EC and higher DOC levels measured in NHS, which are likely to enhance metal solubility through increased ionic strength and complexation. Notably, although total content of all investigated trace elements is higher in HS (Table 2), this does not result in higher leaching for most elements. This observation highlights that leaching behavior is not governed solely by total element content but is strongly influenced by soil-specific factors, including pH, organic matter content, and the chemical binding environment. The stabilization performance for individual trace elements is discussed in detail below based on changes in leachate concentrations across the different binder formulations.

**Copper (Cu) and Nickel (Ni)**

Both copper and nickel exhibit a pronounced increase in leaching following stabilization. This effect is most evident in HS, where Ni concentrations increase from 0.15 ± 0.01 mg/kg^−1^ in the untreated soil to more than 0.75 ± 0.014 mg/kg^−1^ in the 35%A:5%C formulation. Similarly, Cu leaching increases from 0.44 ± 0.065 mg/kg^−1^ to values exceeding 8.6 ± 0.05 mg/kg^−1^ after treatment. These increases are attributed to the formation of soluble metal–hydroxide complexes, such as Cu(OH)_4_^−^ and Ni(OH)_4_^−^, as well as to the development of organo–metal complexes involving dissolved organic carbon (DOC). Such species are stable and mobile under the strongly alkaline conditions generated by the bioash–cement binder, particularly in soils with elevated DOC.

**Figure 5:**
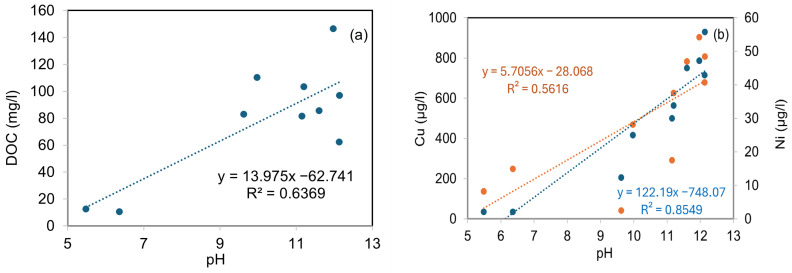
(**a**) Correlation between pH and DOC (t v) and (**b**) between pH and Cu and Ni (t h) in leachate from batch leach tests.

This interpretation is supported by Figure 5, which shows that Cu and Ni concentrations in the leachate increase with increasing pH, while DOC concentrations also rise with pH. The concurrent increase in alkalinity and DOC suggests that high pH promotes the release of organic ligands, which subsequently enhances Cu and Ni mobility through complexation. These trends are consistent with previous studies reporting strong interactions between DOC and transition metals under alkaline conditions [46,47]. These observations highlight a key limitation of the current binder system. A key finding from these results is that stabilization caused a significant increase in Cu and Ni leaching in mixtures with high DOC, especially under highly alkaline conditions. This underscores the strong influence of site-specific soil chemistry on the performance of stabilization.

The observed increase in Cu and Ni leaching under alkaline, DOC-rich conditions suggests that further optimization of the binder system may be required for certain soil types. Potential improvement strategies include the use of additives targeting DOC immobilization (e.g., sorptive carbon-based materials), Fe-based amendments to enhance binding of As and transition metals, and adjustment of cement content or binder composition to better control pH evolution [48,49]. Such approaches could improve the environmental performance of bioash-based stabilization systems while maintaining their mechanical functionality.

**Zinc (Zn) and Cadmium (Cd)**

Zinc and cadmium are effectively immobilized across all binder formulations. In the 35%A:5%C mixture, Zn concentrations decrease from 11.8 ± 1.65 mg/kg to 0.029 ± 0.005 mg/kg in HS and from 37.6 ± 2.5 mg/L to 0.04 ± 0.014 mg/kg in NHS, corresponding to reductions of around 100%. Cd concentrations fall below the analytical detection limit in all stabilized mixtures, with only trace levels detected in a few NHS formulations. The pronounced reduction in Zn and Cd leaching is attributed to their precipitation as low-solubility hydroxide and silicate phases and to adsorption onto binder-derived mineral surfaces [50,51]. Under the highly alkaline conditions generated by the bioash–cement matrix, both elements preferentially form stable solid phases with limited aqueous mobility, resulting in effective immobilization.

**Lead (Pb)**

Lead leaching is significantly reduced after stabilization. In HS and NHS, Pb concentrations decline from 0.12 ± 0.049 mg/kg and 0.1 ± 0.054 mg/kg to 0.03 ± 0.005 mg/kg and 0.01 ± 0.002 mg/kg, respectively, in the 35%A:5%C mixture. This immobilization is attributed to Pb(OH)_2_ precipitation and the formation of low-solubility lead silicates [52]. However, at very high pH levels (e.g., 47.5%A:5%C), Pb concentrations increase slightly again, which is likely due to the formation of soluble Pb-hydroxide complexes [51]. These results highlight the importance of controlling binder composition and pore-solution pH to maintain Pb in sparingly soluble forms and ensure long-term immobilization.

**Arsenic (As)**

Arsenic exhibits contrasting leaching behavior in HS and NHS following stabilization. In HS, arsenic leaching decreases after treatment, consistent with immobilization through the precipitation of calcium hydrogen arsenate (CaHAsO_4_) and calcium arsenate (Ca_3_(AsO_4_)_2_) under alkaline conditions [53]

The elevated calcium availability and high pH generated by the bioash–cement binder promote the formation of these low-solubility calcium–arsenate phases, thereby reducing aqueous arsenic mobility.

In contrast, arsenic concentrations increase in NHS after stabilization. This increase is likely associated with pH-induced desorption of previously sorbed arsenic and increased competition for sorption sites from dissolved organic carbon (DOC), which is higher in NHS. Complexation with organic ligands and reduced affinity for mineral surfaces under strongly alkaline conditions may further enhance arsenic mobility. These divergent responses highlight the strongly soil-specific nature of arsenic behavior and demonstrate that effective arsenic immobilization requires binder strategies tailored to soil chemistry, particularly with respect to organic matter content and DOC release.

**Cobalt (Co) and Chromium (Cr)**

Co leaching decreased in both HS and NHS after treatment, although the effect was less pronounced than for Zn or Cd. Immobilization is likely driven by precipitation as Co(OH)_2_ and surface sorption mechanisms, which are effective at the pH range achieved. Cr showed a slight increase in leaching in NHS after stabilization, while levels remained stable in HS. The oxidation state of Cr was not determined in this study; thus, the mechanisms underlying its mobility remain uncertain. Since Cr(VI) is far more mobile than Cr(III), further speciation would be needed to evaluate long-term risk.

In summary, the effectiveness of the binder formulations followed the general order: Zn > Cd > Pb > Co > As > Cr > Ni > Cu. While the treatment was highly effective for Zn, Cd, and Pb, it was clearly less successful for Cu and Ni, primarily due to DOC-driven complexation under high-pH conditions. This underscores the need for future binder optimization focused on minimizing DOC release or enhancing the capture of organo-metallic complexes. As described in the Section 2, the batch leaching procedure involves specimen crushing, the use of deionized water, and 24 h of agitation, all of which contribute to aggressive extraction conditions. These test parameters are intentionally selected to represent a conservative scenario and are therefore likely to overestimate leaching compared with field conditions. In practical applications, the stabilized material remains compacted and solidified, with limited water exposure and reduced reactive surface area. To complement the batch leaching results and better represent long-term behavior under field-relevant conditions, diffusion testing of intact specimens is performed and discussed in Section 4.4.2.

**Statistical Interpretation of batch leaching Results**

The *p*-values for all recipes are greater than 0.9 for both HS and NHS, suggesting no statistically significant difference in the leaching results between the different binder formulations within each soil type. Additionally, all *p*-values for comparisons between HS and NHS are greater than 0.5, indicating that the leaching results between the two soil types are not statistically significant for any recipe.

#### 4.4.2. Diffusion Leach Tests

**Figure 6:**
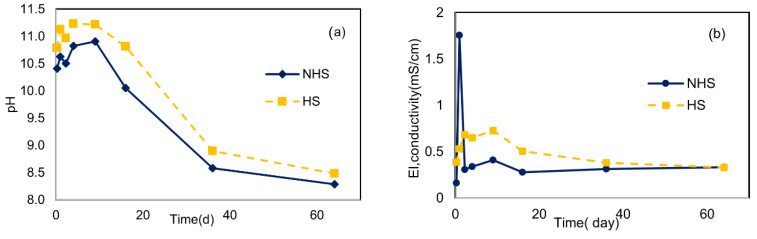

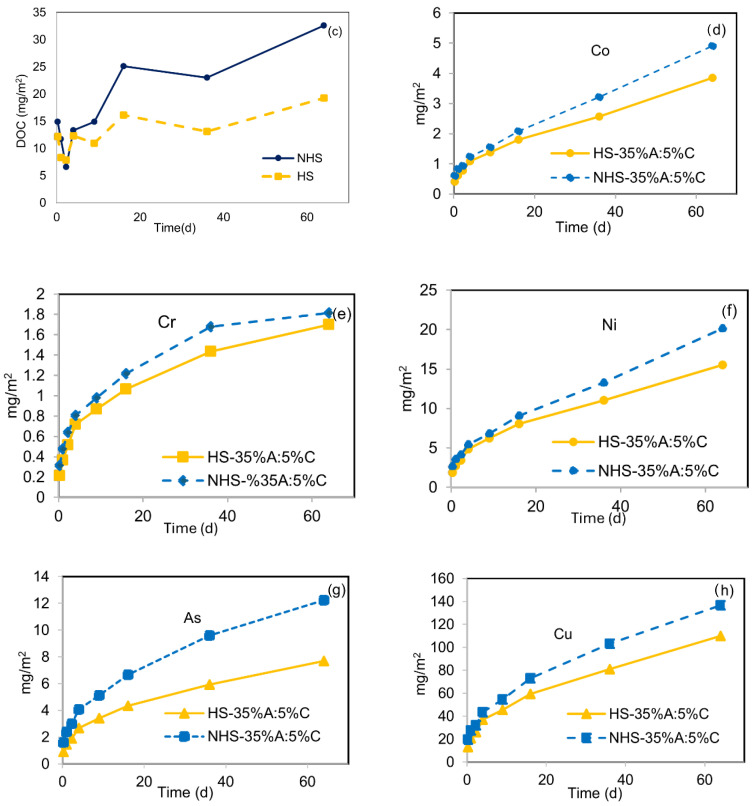
(**a**) pH, (**b**) EC and (**c**) DOC in the leachate after each step, and calculated amounts of leached elements in each step for (**d**) Co, (**e**) Cr, (**f**) Ni, (**g**) As, and (**h**) Cu in diffusion test, for best recipe (35%A:5%C) in NHS, HS.

Diffusion leach tests were conducted using the optimal mixture containing 35% bioash and 5% cement (35%A:5%C) for both HS and NHS. Figure 6 presents the cumulative leaching of selected elements and DOC per unit surface area (mg/m^2^) over a 64-day period, along with trends in pH and EC. elements remained below detection limits throughout the test period, except for very low concentrations of Cd and Pb detected at the final sampling point (day 64). This supports the conclusion that these elements were physically and chemically stabilized within the binder matrix.

In contrast, Cu, Ni, and Co exhibited continuous release over time. Cu showed particularly high mobilization in both soils, with no clear asymptotic trend. DOC release continued throughout the test period, following a consistent upward trend without reaching equilibrium. Ni and Co also showed steady increases, albeit at lower levels. These trends are consistent with weak chemical binding and ongoing transport via mobile DOC species. Cr was detected at low levels in NHS but remained below detection in HS throughout the test. Arsenic was detected in both soils and increased steadily over time, particularly in NHS, where cumulative release exceeded 5 mg/m^2^. Figure 6 also illustrates a progressive increase in EC over time in both soils, along with consistently high pH values (11.4–12.3), which remained stable throughout the test duration. These conditions favor the formation of soluble hydroxide complexes and support continued DOC release, especially in NHS.

The cumulative leaching curves for Cu, Ni, and DOC did not level off, indicating that the elements were not effectively trapped and may remain mobile over time. This was further confirmed by slope analysis following the NEN 7345 method (Table S4). The slope values indicated diffusion-controlled release for all trace elements in HS and for As, Cu, Co, and Ni in NHS. Cr leaching in NHS was categorized as surface wash-off, suggesting that readily soluble, surface-bound fractions were primarily responsible for its release.

Together, these results highlight that while the 35%A:5%C mixture is highly effective in immobilizing Zn, Cd, and Pb over time, it is significantly less effective for Cu, Ni, and As. The persistent release of these elements, particularly in NHS, is attributed to a combination of elevated pH, high DOC levels, and weak binding affinity of these metals to the bioash–cement matrix. These findings underscore the need to consider long-term leaching behavior and binder performance for elements susceptible to complexation and diffusion-driven mobilization.

**Statistical Results for Diffusion Test (HS vs. NHS)**

For all elements tested (Ni, Co, Cr, Cu, As), the *p*-values exceed 0.05, indicating that the differences in leaching behavior between HS and NHS are not statistically significant. The *p*-values for Ni (0.6), Co (0.59), Cr (0.62), Cu (0.52), and As (0.2) suggest that the observed differences are likely due to random variation rather than a true underlying effect. The *p*-value for As (0.2) further supports the conclusion that there is no significant difference in As leaching between HS and NHS.

#### 4.4.3. Leaching Behavior of the Pilot Soil in Laboratory and Field Tests

The leaching behavior of the Pilot soil stabilized with 35% bioash and 5% cement was assessed through laboratory batch testing, solid sampling from the constructed noise barrier, and leachate (surface run-off) monitoring over time.

**In laboratory leaching tests**, the untreated Pilot soil exhibited pH and DOC levels similar to those of HS, but lower than those observed in NHS (pH 5.5 ± 0.2, DOC 14.5 mg/L). However, after stabilization with the same 35%A:5%C binder, the Pilot soil showed a more significant increase in pH (to 12.6 ± 0.02) and EC (to 9.0 ± 0.09 mS/cm) compared to both HS and NHS. Its DOC level (64.5 ± 1.97 mg/L) approached the high value observed in NHS (up to 146.5 mg/L). In contrast to the HS and NHS, where Zn, Cd, and Pb were consistently well retained, the Pilot soil demonstrated strong retention of Zn and Cd as well, but increased solubility of Pb, As, Cu, Ni, and Cr. This outcome closely resembles the less favorable response seen in NHS, suggesting that the Pilot soil shares key chemical features, particularly high DOC and a more reactive organic fraction, that reduce the binder’s effectiveness for certain elements.

While Zn and Cd concentrations fell below detection limits after treatment, several other elements exhibited increased leaching. Pb concentrations rose from 0.2 mg/kg to 1 mg/kg, likely due to the formation of soluble hydroxide species under high pH and reduced sorption. Arsenic increased modestly (~3%), possibly reflecting pH-driven conversion from As(V) to the more mobile As(III). Cu, Ni, and Cr leaching also increased substantially, which is attributed to the formation of soluble metal–organic complexes promoted by elevated DOC and alkaline conditions. Although this binder formulation achieved the highest UCS among all tested soils (see Figure 3b), it failed to sufficiently stabilize several elements in the Pilot soil. These results emphasize the influence of site-specific factors, particularly DOC and soil mineralogy, on stabilization outcomes (Table 3).

**Comparison of leaching behavior in laboratory tests for different types of soil**

The results clearly indicate that soil chemistry, particularly DOC levels, plays a pivotal role in determining the stabilization and immobilization efficiency of the bioash-cement binder system. DOC content in soils is a critical factor influencing the leaching behavior of trace metals, especially Cu and Ni, due to the formation of organic–metal complexes that increase metal solubility. In soils with high DOC (such as NHS and Pilot soils), leaching of Cu and Ni was more pronounced, as organic ligands enhance the mobility of these metals under high-pH conditions. The complexation between DOC and metals leads to the formation of soluble complexes, reducing the binder’s effectiveness for certain metals. Therefore, further optimization of the binder, such as reducing DOC release or adding agents to improve metal retention, is essential for these soils. In contrast, soils with lower DOC (like HS) showed more effective performance with the bioash-cement binder system, as the precipitation of metal hydroxides and sorption onto binder phases (like C-S-H gels) significantly reduced metal solubility and mobility. In these soils, the high calcium content in the binder played a key role in precipitating metals like Zn and Cd, while iron oxides and other phases provided adsorption sites for Pb and As.

These findings suggest that the performance of bioash-cement binders can be generalized by considering soil-specific factors such as DOC levels, pH, and mineral composition. Higher DOC content leads to greater metal leaching, particularly for Cu and Ni, whereas lower DOC content enhances the binder’s effectiveness in immobilizing metals. To optimize the system for soils with high DOC, incorporating additives like iron-based amendments or carbonaceous materials (such as activated carbon) could help limit DOC release and improve metal retention.

**Figure 7:**
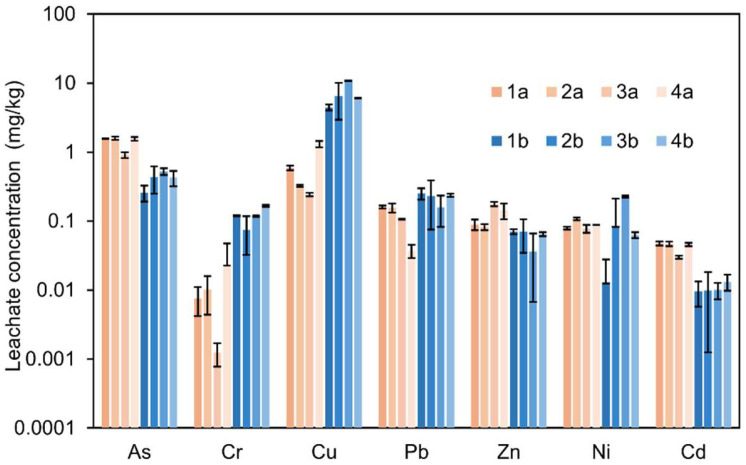
Leaching from surface (a; 0–5 cm) and deeper (b; 10–20 cm) samples from the noise barrier taken two months after construction.

**In field samples** taken from the constructed noise barrier two months after completion, depth-dependent leaching behavior was observed (Figure 7). The surface layer (0–5 cm) exhibited lower pH (8–9), EC (500–2000 µS/cm), and DOC (5–15 mg/L), and correspondingly lower leaching of Cr, Cu, and Pb—likely due to sorption and precipitation under mildly alkaline conditions. In contrast, Zn, Cd, and As were more mobile in this layer, potentially due to less stable C-S-H phases near neutral pH and partial oxidative dissolution of arsenic [50,53].

The deeper layer (10–20 cm) had much higher pH (12.3–12.8), EC (2000–6700 µS/cm), and DOC (22–66 mg/L), and exhibited increased leaching of Cu, Pb, and Cr. This is attributed to the formation of hydroxo-complexes (e.g., Cu(OH)_4_^2−^, Cr(OH)_3_^−^) and DOC-mediated mobilization under strongly alkaline conditions [46,47]. The highest DOC concentrations were observed in deeper barrier samples (notably 3b and 4b), which coincided with elevated leaching of Cu and Ni. This supports the hypothesis that DOC plays a key role in their mobilization under strongly alkaline conditions.

Nevertheless, Zn, Cd, and As leaching remained low in the deeper zone, consistent with their stabilization via precipitation as zinc silicate and cadmium hydroxide at high pH [51,52]. These depth-dependent results illustrate how variable pH and DOC within the barrier can differently affect the retention of specific elements.

**Trace Element Content in Untreated Stabilized Noise Barrier Material: Post-Treatment Analysis**

The lower part of Table 2 summarizes the total trace element concentrations measured in the untreated Pilot soil, the binder materials used in the pilot-scale application, and the stabilized material sampled from the constructed noise barrier. Post-treatment samples were collected from two depths within the barrier, namely the surface layer (0–5 cm, layer a) and a deeper layer (10–20 cm, layer b).

Analysis of the stabilized barrier material presented in Table 4 indicated lower total concentrations for most trace elements relative to the untreated Pilot soil. Zinc was the only element showing slightly increased concentrations, approximately 1.5 times higher than in the untreated soil; however, subsequent leaching tests (Section 4.4.1) demonstrated limited zinc mobility. Mean concentrations in the surface and deeper layers were generally comparable, although slightly lower copper and nickel concentrations were observed in the deeper layer. With the exception of arsenic, all trace element concentrations in the stabilized material were below the LSL thresholds. The observed reductions in total concentrations are attributed primarily to dilution resulting from the incorporation of binder materials, rather than to removal or loss of contaminants through leaching.

**Figure 8:**
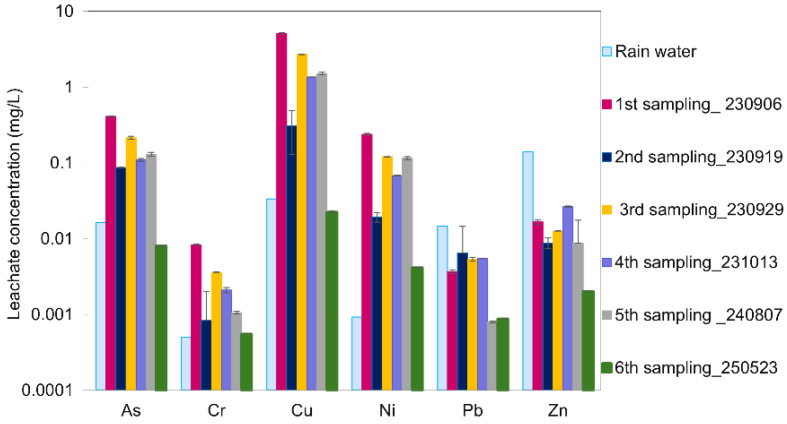
Unfiltered leachate concentrations of elements and rainwater from the pilot test with the noise barrier over six sampling events across two years.

**Leachate monitoring over a nearly two-year period** further demonstrated the evolving chemical stability of the stabilized structure (Figure 8, Table 5). Initial leachate samples showed high pH (12.3) and EC (7.2 mS/cm), along with elevated concentrations of As (0.41 mg/L) and Cu (5.10 mg/L). Over time, pH and EC gradually declined, reaching 10.2 and 2.2 mS/cm, respectively, by August 2024. Trace metal concentrations also decreased. The final sampling round, conducted in May 2025, showed that arsenic and copper levels had dropped further to 0.008 mg/L and 0.02 mg/L, respectively. Ni showed a moderate but steady decline, while Pb and Zn remained consistently low. Cd and Cr were below detection limits in nearly all samples. These trends suggest progressive stabilization, likely due to aging processes such as carbonation, mineral transformations, or reduced DOC availability. The observed decrease in pH and EC over time is consistent with carbonation processes in Ca-rich cementitious systems. Carbonation occurs when calcium hydroxide (Ca(OH)_2_) reacts with atmospheric CO_2_, forming calcium carbonate (CaCO_3_). This reaction leads to a gradual reduction in pore solution alkalinity and ionic strength, which is reflected in the declining EC values and the improved long-term stabilization of leached elements [54]. These trends suggest ongoing aging processes within the stabilized material, enhancing chemical stability under field conditions. Specifically, these aging effects likely involve carbonation, mineral transformations, and/or the reduced availability of DOC. The progressive immobilization of trace elements can be attributed to several aging mechanisms associated with cementitious binders. For instance, carbonation promotes the formation of secondary carbonate phases, which can facilitate the co-precipitation or incorporation of metals such as Cu and Ni. Additionally, iron-bearing phases present in both the binder and the soil act as strong sorption sites for metals, particularly for oxy-anions like arsenic (As) [55,56]. At the same time, the continued evolution and densification of C-S-H-type gels reduce pore connectivity, limiting diffusive transport of contaminants. These combined processes provide a mechanistic explanation for the improved long-term leaching performance observed in the pilot-scale trials compared to the earlier laboratory result.

Rainwater collected during the same period exhibited a near-neutral pH (5.8) and very low electrical conductivity (0.01 mS/cm), but trace metal concentrations were markedly elevated. Compared to background values for Swedish precipitation [57] the measured concentrations of Pb, Cd, As, Cr, and Cu exceeded natural levels by several orders of magnitude. Additionally, when compared to recent deposition data from Holmsvattnet near Rönnskär [58], the rainwater at the pilot site showed comparable or even higher concentrations for several metals (Table S5). This indicates that precipitation in the area was already contaminated by atmospheric deposition, likely influenced by historic and ongoing emissions from Boliden’s smelting operations. Rather than acting as a neutral infiltration source, rainwater under these conditions functions as a carrier of diffuse pollution and must be considered when evaluating field leaching behavior and the long-term performance of the stabilization system (see also Table 5).

Together, these results demonstrate that while the binder system was only partially effective under aggressive laboratory conditions, it performed more favorably under field conditions—particularly over time. The evolving geochemistry within the stabilized structure, coupled with the notable influence of contaminated precipitation, highlights the complexity of real-world applications. These findings emphasize not only the importance of long-term monitoring but also the need to account for site-specific external factors such as rainwater composition. Local geochemical conditions—especially pH and DOC—play a central role in determining the element-specific performance of stabilization strategies and should be carefully considered in future binder development and field-scale implementation.

**Laboratory-Scale Results vs. Pilot-Scale Results**

While the laboratory trials provided controlled conditions for testing the bioash-cement binder system, the pilot-scale field trials introduced several real-world factors that influenced the results. These discrepancies highlight the variability that can occur when scaling up from laboratory to field applications. The key factors that may explain these differences include compaction variability, moisture content fluctuations, and environmental exposure, which were not present or tightly controlled in the laboratory settings.

1. Unconfined Compressive Strength (UCS): Laboratory trials consistently yielded higher UCS values compared to the pilot-scale trials. This difference can be attributed to the greater control over moisture content and compaction during laboratory testing, which led to more uniform binder distribution and stronger material formation. In contrast, the pilot-scale trials involved larger volumes of soil, with less uniform moisture content and compaction methods, which likely resulted in a more heterogeneous material matrix. Despite this, the binder system showed satisfactory mechanical strength at the pilot scale, though not as high as the laboratory results.
2. Leaching Behavior: Leaching tests conducted during the laboratory trials showed higher concentrations of trace metals in the leachate compared to the field trials. This discrepancy can be explained by the long-term carbonation and mineral transformation processes occurring in the field. Field conditions, such as exposure to atmospheric CO_2_ and fluctuating environmental conditions, contributed to the formation of secondary phases like metal carbonates and hydrated compounds, which reduced contaminant mobility over time. In the laboratory, these processes were not as pronounced due to the controlled curing environment.
3. Environmental Factors: While the laboratory-scale trials allowed for precise control over the binder-water interactions, the pilot-scale trials were influenced by factors such as rainfall, temperature fluctuations, and soil heterogeneity. These environmental factors likely impacted both binder hydration and the stabilization of contaminants, leading to variations in performance when compared to the laboratory results. However, these field conditions better reflect the actual behavior of the bioash-cement binder system under real-world applications, providing valuable insights into its long-term stability and environmental performance.
4. Implications for Real-World Applications: The differences between the laboratory-scale and pilot-scale results highlight the importance of considering site-specific conditions when applying bioash-cement binder systems in the field. While laboratory trials are critical for binder optimization, pilot-scale trials provide more reliable data for scaling up and long-term performance evaluation. These findings underscore the necessity of further field validation to ensure that the binder formulation maintains its effectiveness in larger-scale, more variable conditions.

## 5. Synthesis and Implications

This study evaluated the stabilization/solidification (S/S) of metal-contaminated soils using a bioash–cement binder across both laboratory and field settings. The selected binder formulation (35% bioash, 5% cement) effectively reduced the mobility of key elements such as Zn and Cd. However, mechanical performance, while acceptable for the intended low-load application, remained modest and comparable to that of compacted clays or lightly stabilized soils. Cu and Ni were not reliably immobilized, particularly under conditions of elevated pH and DOC, highlighting that meeting strength criteria does not necessarily ensure element-specific chemical stabilization.

The variation in treatment success across HS, NHS, and Pilot soil already in laboratory testing demonstrated the strong influence of site-specific soil chemistry and matrix characteristics, including the role of organic matter and DOC in controlling metal speciation and mobility. Field implementation reinforced this sensitivity, while also introducing practical constraints: uneven mixing and variable compaction during construction likely contributed to reduced performance in the upper parts of the structure. Leachate trends improved over time, suggesting that aging processes (e.g., carbonation, ongoing mineral transformations, or reduced DOC release) can further enhance immobilization, but also emphasizing that stabilization may evolve gradually rather than being fully achieved immediately after placement.

The influence of locally contaminated rainwater—containing trace metals well above Swedish background levels—further complicates interpretation of field leaching, since precipitation represents a continuous contaminant input rather than a chemically neutral infiltrating fluid. In addition, long-term biological colonization should be considered as a secondary but potentially relevant process in field performance: vegetation establishment and root development can alter pore structure, macroporosity, and preferential flow pathways over time, thereby affecting infiltration patterns and solute transport. Evidence from structural (CT-based) studies of root–soil system development (e.g., [59]) indicates that root network evolution can increase macropore connectivity and reorganize soil structure over decadal scales; while the climatic setting differs, the underlying mechanism—root-driven modification of pore networks—supports considering ecological and biological factors when interpreting long-term hydraulic and leaching behavior in field S/S applications.

From a technical perspective, future improvements should include more robust, full-scale compaction procedures (e.g., heavy vibratory rollers, as commonly applied in road and embankment construction and stricter lift control) and where feasible, better-controlled pre-blending of dry binders and soil before water addition to reduce heterogeneity. Binder composition may also need modification for elements sensitive to DOC (notably Cu and Ni). One practical option is the incorporation of iron-based amendments (e.g., Fe-oxides or zero-valent iron) which can provide additional sorption capacity and/or reduce DOC-driven metal mobilization, thereby improving retention of metals that are otherwise poorly controlled in high-pH, DOC-rich porewaters.

## 6. Comparison with Conventional Cement-Based Binder Systems

To place the performance of the bioash–cement composite binder in context, the results are compared with those reported for conventional cement-only stabilization systems commonly used for soil treatment. Cement-based binders are well known to provide rapid strength development and effective immobilization of several divalent metals; however, they typically require higher cement contents, which increase environmental impact and cost.

In terms of mechanical performance, the optimized bioash–cement formulation containing 35 wt.% bioash and 5 wt.% cement achieves unconfined compressive strength (UCS) values in the range of 700–1000 kPa under laboratory conditions, depending on soil type and compaction energy. These values are comparable to those reported for soils stabilized with 8–12 wt.% Portland cement, which commonly yield UCS values between 500 and 1500 kPa for fine-grained soils. The results therefore indicate that partial replacement of cement with bioash can maintain adequate mechanical performance while significantly reducing cement demand [60,61].

With respect to leaching behavior, conventional cement-only systems typically achieve effective immobilization of Zn, Cd, and Pb through pH elevation and the formation of low-solubility hydroxide and carbonate phases. The bioash–cement binder exhibits similar immobilization efficiency for these elements, with reductions exceeding 99% for Zn and near-complete removal of Cd from the leachate. This demonstrates that bioash does not compromise the stabilization of these metals when used as a supplementary binder component [62,63].

An important distinction between the bioash–cement composite and cement-only binders is the increased DOC release observed when bioash is incorporated. While this DOC contributes to enhanced Cu and Ni mobility, it also highlights a key parameter for future binder design. Strategies such as ash pre-treatment, blending with low-organic mineral additives, or incorporation of sorptive phases may mitigate DOC-related effects while preserving the benefits of cement reduction.

Overall, the comparison demonstrates that the bioash–cement binder achieves mechanical and environmental performance comparable to conventional cement-based stabilization systems, despite using substantially lower cement contents. From a materials engineering perspective, the results indicate that bioash can function as an effective supplementary binder component, enabling the development of lower-carbon cementitious materials for soil stabilization and other low-load construction applications. Further optimization is required to address DOC-driven metal mobility, but the performance achieved in this study is consistent with, and in several aspects comparable to, that of traditional cement-only recipes.

## 7. Conclusions

This study highlights the potential of bioash-based binders as a sustainable alternative for stabilizing metal-contaminated soils, offering significant environmental benefits by reducing cement consumption and lowering CO_2_ emissions. The results show that bioash-cement mixtures are effective in immobilizing key contaminants like Zn, Cd, and Pb, making them suitable for low-load applications, such as noise barriers. However, the effectiveness of this binder system is highly influenced by soil-specific factors, particularly dissolved organic carbon (DOC) content. Soils with high DOC, such as NHS and Pilot soils, exhibited increased leaching of metals like Cu and Ni, highlighting the challenges posed by organic matter in the stabilization process.

To further enhance the performance of bioash-based binders, future research should focus on optimizing binder compositions to minimize DOC release and improve metal retention. Potential improvements include incorporating additives, such as iron-based amendments or carbonaceous materials, to reduce the solubility of metal–organic complexes and support long-term immobilization. Additionally, refining the binder’s composition to address the mobility of arsenic in high-DOC soils remains a critical area for optimization.

These findings emphasize the importance of considering soil chemistry and environmental conditions when developing bioash-based stabilization systems. Long-term field monitoring over two years demonstrated significant improvements in contaminant retention, supporting the system’s long-term efficacy. This ongoing evaluation will be key to guiding future advancements and expanding the applicability of bioash-based stabilization for a broader range of contaminated soils.

## Figures and Tables

**Table 1:** Summary of the soil types and their abbreviations, regulatory classifications, particle size distributions, and applications in laboratory and field experiments.

Soil-ID	Abbreviation	Classification	Dominant Contaminants	Particle Size Distribution of Soils
Hazardous soil	HS	Hazardous	Laboratory tests	Sandy
Non-Hazardous soil	NHS	Non-Hazardous	Laboratory tests	Sandy
Näsudden soil	Pilot soil	Non-Hazardous	Laboratory and field	Sandy-Silt mix

**Table 2:** TS and vs. (%), TOC (mg/L), and trace element concentrations (mg/kg) in untreated HS and NHS and pilot soil used in the pilot test of stabilized material. LOD: Limit Of Detection.

Sample	TS	VS	TOC	As	Cu	Ni	Cr	Pb	Zn	Cd
Sensitive land (SL)				10	80	40	80	50	250	0.5
Less sensitive land (LSL)				25	200	120	150	180	500	15
Hazardous waste [40]				1000	2500		10,000	2500	2500	100
	Untreated HS and NHS in pre-trial recipe development, 2022									
HS	81.6	5.6	10.48	168	266	49	181	548	2687	17.5
NHS	76.7	9.0	12.6	102	160	23	132	240	769	<LOD
	Bioash (2022: pre-trial recipe development; 2023: used in pilot trials)									
Bioash 2022	92.0	4.1	2.36	47	81	120	87	74	565	<LOD
Bioash 2023	99.8	0.3	3.1	21	143	92	70	47	670	<LOD
Cement	99.8			<LOD	<LOD	<LOD	<LOD	31.25	1026	<LOD
	Untreated pilot soil (excavated June 2023)									
Pilot soil	91.0	4.1	9.4	192	272	<LOD	100	370	214	19.5

**Table 3:** The concentrations (mg/kg) of elements in the leachate from untreated and treated pilot soil are presented below. The treated pilot soil is a mixture of 60% pilot soil, 35% bioash, and 5% cement (60%Pilot soil:35%A:5%C, with a liquid-to-solid ratio (L/S) of 10. LOD: Limit of Detection.

Elements	As	Cr	Cu	Ni	Pb	Zn	Cd
Untreated Pilot Soil	0.58 ± 0.07	0.005	0.63 ± 0.07	0.079	0.2 ± 0.03	8.39 ± 0.29	0.088
60% Pilot soil:35% A:5%C	0.60	0.034	1.09	0.75	1.04	0.09	<LOD

**Table 4:** Pilot soil used in the pilot test stabilized with 35% bioash and 5% cement and water, soil taken from the loaf two months after construction (1–4 a and b; September 2023) as well as bioash and cement used for stabilization. LOD: Limit Of Detection.

	Stabilized Soil Samples from the Pilot Test (1–4 a and b; September 2023)									
Sample	TS	VS	DOC	As	Cu	Ni	Cr	Pb	Zn	Cd
1a	80.5	5.3	8.4	61	108	<LOD	55	129	422	<LOD
2a	72.4	5.0	5.2	63	95	<LOD	46	116	374	<LOD
3a	73.4	4.7	5.5	65	94	<LOD	98	108	273	<LOD
4a	86.7	6.2	15.5	67	103	<LOD	47	132	305	<LOD
1b	74.5	4.8	34.4	51	99	<LOD	31	94	307	17.6
2b	75.3	4.2	22.8	54	93	<LOD	36	125	356	<LOD
3b	76.0	4.8	66.5	47	114	<LOD	97	117	400	<LOD
4b	77.4	4.8	52.2	64	113	<LOD	46	125	404	<LOD
AVE a	78.2	5.3	8.7	56	104	<LOD	70	112	351	<LOD
AVE b	75.9	4.7	44.1	62	101	<LOD	44	125	360	<LOD

**Table 5:** pH, EC, and Metal Concentrations (mg/L) in unfiltered leachate and in Rainwater.

	Leachate						Rain Water
Date	6 September 2023	19 September 2023	29 September 2023	13 October 2023	7 August 2024	23 May 2025	23 October 2023
pH	12.3	9.4	11.1	10.5	10.2	7.9	5.8
EC, mS/cm	7.2	1.19	2.81	2.25	2.16	0.34	0.01
As	0.411 ± 0.008	0.087	0.215 ± 0.009	0.110 ± 0.005	0.133 ± 0.007	0.008	0.016
Cd	0.0002	0.000055	0.000085	0.00007	0.00002	0.00005	0.00014
Cr	0.0005	0.0083 ± 0.001	0.00083	0.0036	0.0021	0.0005	0.0005
Cu	5. 095 ± 0.15	0.31 ± 0.18	2.7 ± 0.02	1.36	1.57 ± 0.06	0.02	0.033
Ni	0.238 ± 0.007	0.019 ± 0.002	0.121	0.06 ± 0.001	0.117 ± 0.004	0.004	0.00092
Pb	0.003	0.006 ± 0.008	0.005	0.005	0.001	0.0008	0.014
Zn	0.016 ± 0.001	0.008 ± 0.001	0.012	0.026	0.009 ± 0.008	0.002	0.14

## Data Availability

The original contributions presented in this study are included in the article/Supplementary Materials. Further inquiries can be directed to the corresponding author.

## Supplementary Materials

The following supporting information can be downloaded at: https://www.mdpi.com/article/10.3390/ma19040790/s1, Figure S1. Particle size distribution of Hazardous Soil (HS), Non-Hazardous Soil (NHS), Pilot Soil, and Bioash. The graph shows the cumulative percentage of particles passing through each particle size, highlighting the distinct differences in particle size distribution; Figure S2. (a) Cement was added to the contaminated soil. (b) Bioash was weighed in a scoop and then mixed with the contaminated soil. (c) Contaminated soil, cement and bioash were applied in several layers and premixed with a shovel. (d) At the site where the noise barrier was to be built, the masses were mixed with water in a mixing pit. (e) The masses were finally mixed with an aluminum scoop in connection with placing them in place in the noise barrier. (f) Loaf in September 2023, 3 months after solidification. Moss has started to grow on the embankment. Photos were taken by Lale Andreas; Figure S3. (a) A tarpaulin under the noise barrier collects leachate, which flows by gravity into a container. (b) Sampling of leachate from the container at the noise barrier; Figure S4. (a,b) Samples were collected for ICP, DOC, and TOC test.; Table S1. Chemical composition of Pilot soil, bioash, and cement; Table S2. Optimum water content and dry density for different bioash–cement binder mixtures: 35%A:5%C, 47.5%A:5%C, for P-HS and P-NHS, and 35%A:5%C for the Pilot soil; Table S3. pH, EC and DOC in leaching test for NHS and HS before and after stabilization; Table S4. Diffusion-controlled mechanism of trace element leaching; Table S5. Comparison of trace metal concentrations in unfiltered rainwater collected at the pilot site with natural background values and estimated concentrations from precipitation at Holmsvattnet (2021/22), near the Rönnskär industrial area. All concentrations are in µg/L.
