# Supplementary material for: Bioash-Based Stabilization/Solidification for Heavy Metal(oid) Soil Remediation: A Case Study in Northern Sweden
Source: Materials (Basel). 2026 Feb 18;19(4):790. doi: 10.3390/ma19040790 (PMC12941421; doi:10.3390/ma19040790)
Supplement: Supplementary file 1 [file materials-19-00790-s001.zip › materials-4127448-supplementary.pdf]

materials

MDPI

Supplementary

Bioash-Based Stabilization/Solidification for Heavy Metal(oid) Soil Remediation: A Case Study in Northern Sweden

Sepideh Gholizadeh Khasevani [1,*], Ivan Carabante [1], Josef Bjuhr [2] and Lale Andreas [1]

[1] Waste Science and Technology, Department of Civil, Environmental and Natural Resources Engineering, Luleå University of Technology, 97187 Luleå, Sweden; ivan.carabante@ltu.se (I.C.); lale.andreas@ltu.se (L.A.)
[2] AFRY, Infrastructure AB, Frösundaleden 2A, 16970 Solna, Sweden; josef.bjuhr@afry.com
* Correspondence: sepideh.gholizadeh.khasevani@ltu.se

Particle size distributions of soils and bioash

The particle size distributions of the soils (HS, NHS, and Pilot Soil) and bioash are presented in the figure above. The curve illustrates the percentage of material passing through each particle size, showing distinct differences in the particle size distributions of the four materials.

HS (Hazardous Soil): This soil exhibits a relatively steep curve, indicating that a majority of the material falls within the coarser fractions, with a substantial amount of particles in the sand size range (greater than 2 mm).

- NHS (Non-Hazardous Soil): Similarly to the HS, the NHS also shows a high percentage of coarse particles, though there is a slightly more gradual curve, indicating a more uniform particle size distribution compared to HS.
- Pilot Soil: This sample shows a mixture of fine and coarse particles, with a more gradual distribution than the HS and NHS, suggesting a balance between sand- and silt-sized particles.
- Bioash: The bioash has a highly uniform particle size, with almost all the material passing through the finer sieves, reflecting its powdery, fine nature. The particle size distribution of bioash is concentrated in the silt and clay ranges, with a steep curve indicating fine particle sizes.

These results indicate that the bioash is significantly finer than the soils, which could impact its reactivity in the stabilization process, particularly for contaminant immobilization.

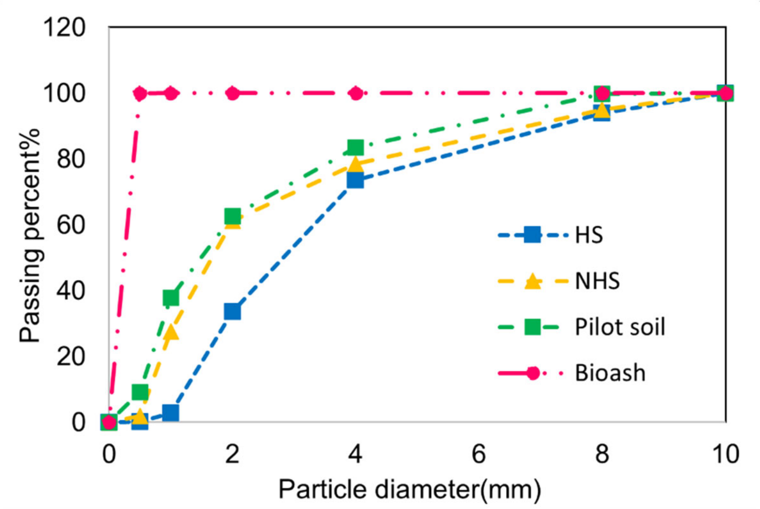

Figure S1. Particle size distribution of Hazardous Soil (HS), Non-Hazardous Soil (NHS), Pilot Soil, and Bioash. The graph shows the cumulative percentage of particles passing through each particle size, highlighting the distinct differences in particle size distribution.

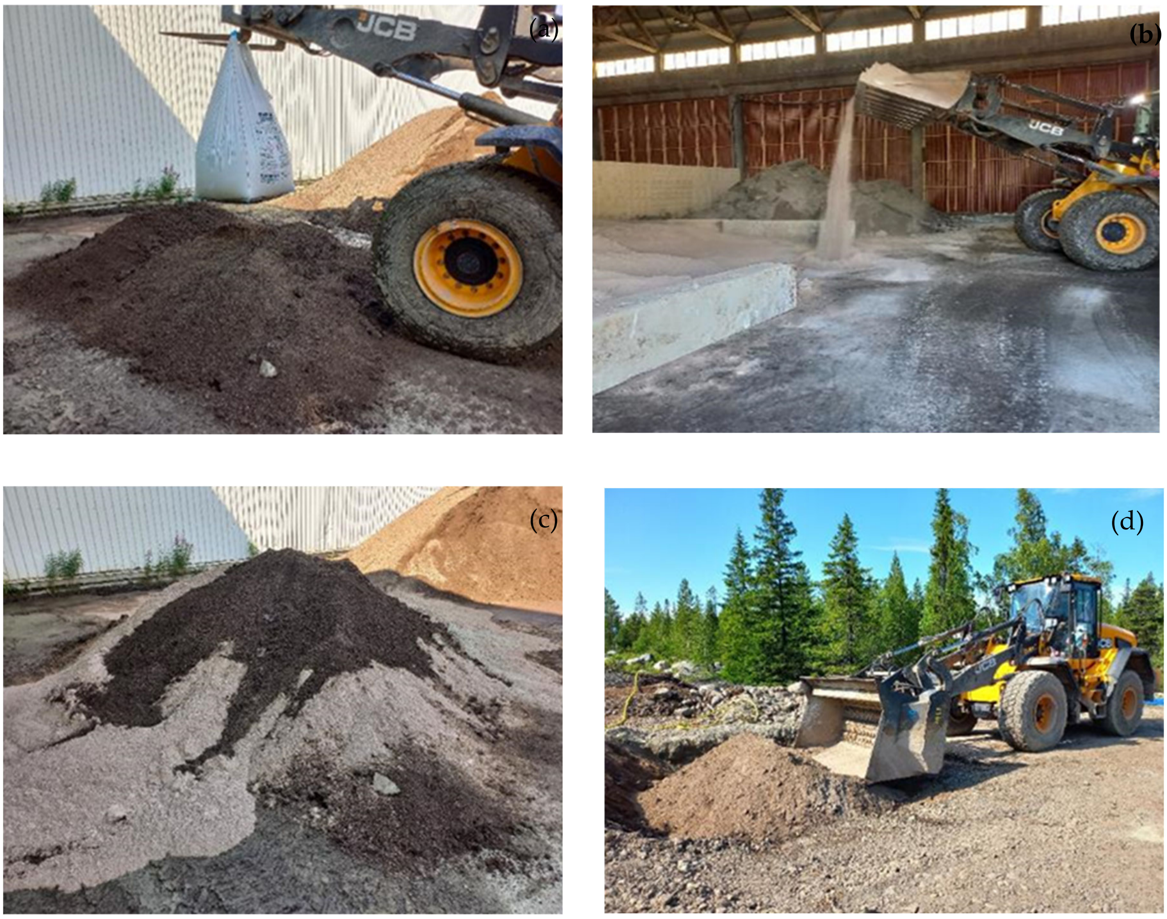

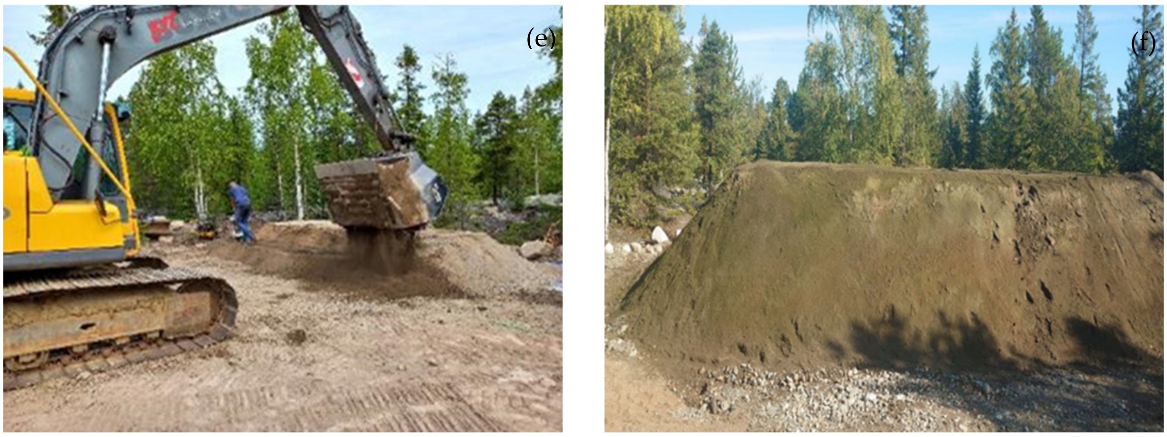

Figure S2. (a) Cement was added to the contaminated soil. (b) Bioash was weighed in a scoop and then mixed with the contaminated soil. (c) Contaminated soil, cement and bioash were applied in several layers and premixed with a shovel. (d) At the site where the noise barrier was to be built, the masses were mixed with water in a mixing pit. (e) The masses were finally mixed with an aluminum scoop in connection with placing them in place in the noise barrier. (f) Loaf in September 2023, 3 months after solidification. Moss has started to grow on the embankment. Photos were taken by Lale Andreas.

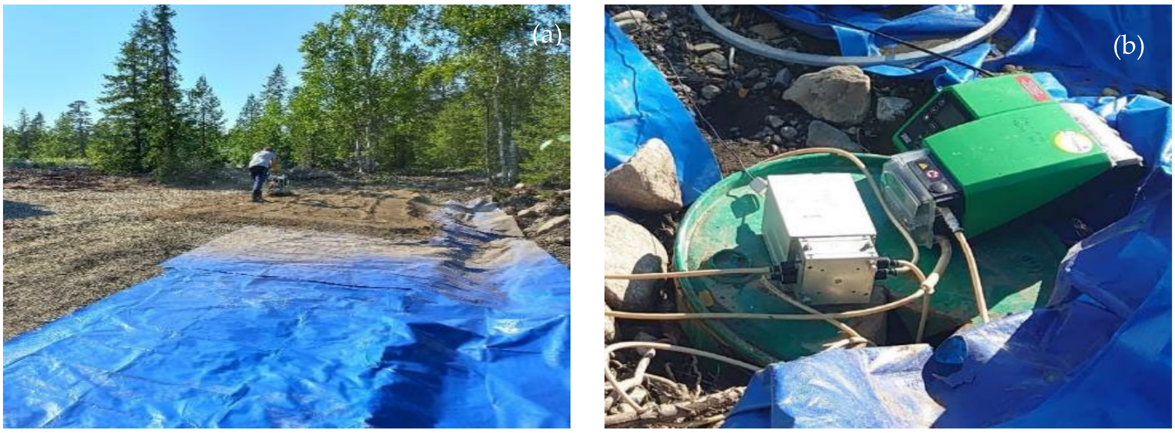

Figure S3. (a) A tarpaulin under the noise barrier collects leachate, which flows by gravity into a container. (b) Sampling of leachate from the container at the noise barrier.

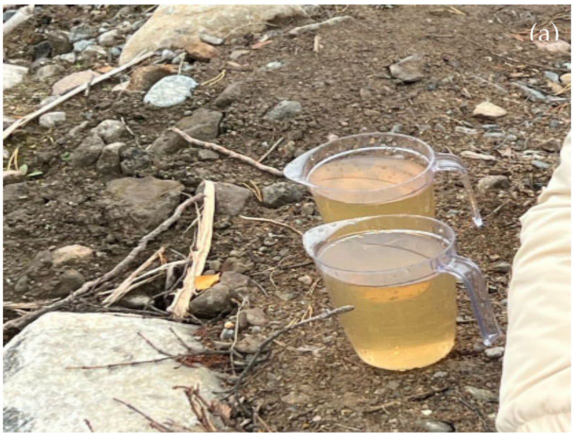

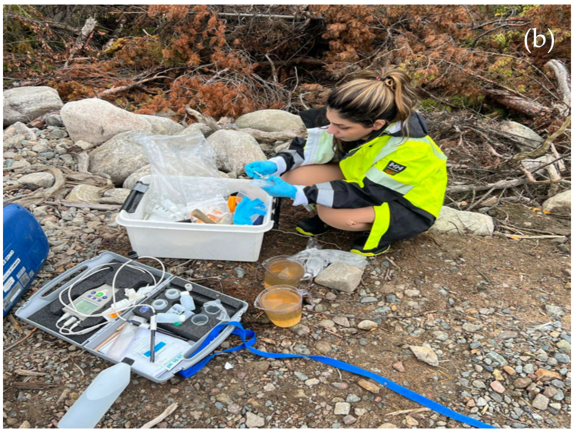

Figure S4. (a,b) Samples were collected for ICP, DOC, and TOC test.

Table S1. Chemical composition of Pilot soil, bioash, and cement.

Sample	SiO_2	Al_2O_3	Fe_2O_3	CaO	MgO	Na_2O	SiO_2+Al_2O_3+ Fe_2O_3	CaO/ SiO_2
Description	%	%	%	%	%	%		
Pilot Soil	67.8	12.25	3.67	2.47	1.1	3.45	83.72	0.036
Bioash	24.1	5.96	2.81	33.7	3.88	1.22	32.87	1.39
Cement	21.2	3.4	4.1	63.3	2.2	0.18	28.7	2.9

Table S2. Optimum water content and dry density for different bioash–cement binder mixtures: 35%A:5%C, 47.5%A:5%C, for P-HS and P-NHS, and 35%A:5%C for the Pilot soil. (*A = bioash; C = cement*).

Sample	Optimum water content %	Maximum dry density (g/cm³)
HS-A35:C5	10	1.36
NHS-A35:C5	10	1.25
HS-A47.5:C5	21	1.32
NHS-A47.5:C5	21	1.23
Pilot soil- A35:C5	15	1.57

Table S3. pH, EC and DOC in leaching test for NHS and HS before and after stabilization.

	pH	El. Cond. (mS/cm)	DOC (mg/l)
HS	6.4	4.1	10.5
HS-A35	11.2	1.9	81.7
HS-A50	11.6	2.0	85.5
HS-A35:C5	12.1	4.3	96.9
HS-A47.5:C5	12.3	3.6	62.3
NHS	5.5	7.0	12.6
NHS -A35	9.6	1.3	83.0
NHS -A50	11.2	1.3	103.4
NHS - A35:C5	12.1	2.2	146.5
NHS-A47.5:C5	12.3	3.0	110.4

https://doi.org/10.3390/xxxxx

Table S4. Diffusion-controlled mechanism of trace element leaching.

Trace elements	Slop- HS	Mass transfer mechanism	Slop- NHS	Mass transfer mechanism
As	0.37	DF	0.38	DF
Cu	0.38	DF	0.35	DF
Cr	0.37	DF	0.32	SW
Co	0.39	DF	0.37	DF
Ni	0.38	DF	0.36	DF

Table S5. Comparison of trace metal concentrations in unfiltered rainwater collected at the pilot site with natural background values and estimated concentrations from precipitation at Holmsvattnet (2021/22), near the Rönnskär industrial area. All concentrations are in µg/L.

Element	Rainwater at Pilot Site	Natural Background*	Estimated Rainwater Holmsvattnet (2021/22)
Zn	1.6	< 3	61.4
Cu	23	< 1	16.4
Pb	140	< 0.005	12.4
Cd	< 55	< 0.005	0.58
As	16	< 0.2	2.6
Ni	14	< 0.5	1.2
Cr	33	< 0.2	0.44

Footnote:

Natural background values are taken from SLU monitoring data and from Naturvårdsverket [57], Rapport 4919: Atmosfärdeposition av metaller – bakgrundshalter och trender. Estimated concentrations for Holmsvattnet are derived from open field deposition values (2021/22) in IVL Report C 762 [58] (Nedfall och avrinning av metaller, svavel och kväve i närheten av Rönnskärsverken) and calculated based on an annual precipitation of 635 mm. Concentrations at the pilot site are based on unfiltered rainwater samples collected on 2023-10-23 (see Table 5). Deposition values from Holmsvattnet were estimated visually from Figures 4–6 of the IVL report.

References

[57] Naturvårdsverket (Swedish Environmental Protection Agency). *Naturvårdsverket Bedömningsgrunder För Sjöar Och Vattendrag—Bilaga A: Metaller*, 4th ed.; Naturvårdsverket: Stockholm, Sweden, 2007.

[58] IVL Svenska Miljöinstitutet. *Nedfall och avrinning av metaller, svavel och kväve i närheten av Rönnskärsverken*; Report C 762; IVL Svenska Miljöinstitutet: Stockholm, Sweden, 2023.
